# IL‐27 Rα^+^ cells promoted allorejection via enhancing STAT1/3/5 phosphorylation

**DOI:** 10.1111/jcmm.15700

**Published:** 2020-08-06

**Authors:** Shanshan Zhao, Ting Liang, Chao Zhang, Dai Shi, Wen Jiang, Chen Su, Guihua Hou

**Affiliations:** ^1^ Key Laboratory for Experimental Teratology of the Ministry of Education and Biomedical Isotope Research Center School of Basic Medical Sciences Cheeloo College of Medicine Shandong University Jinan China

**Keywords:** allorejection, apoptosis, IFN‐γ, IL‐10, IL‐27Rα, STAT

## Abstract

Recently, emerging evidence strongly suggested that the activation of interleukin‐27 Receptor α (IL‐27Rα) could modulate different inflammatory diseases. However, whether IL‐27Rα affects allotransplantation rejection is not fully understood. Here, we investigated the role of IL‐27Rα on allorejection both in vivo and in vitro. The skin allotransplantation mice models were established, and the dynamic IL‐27Rα/IL‐27 expression was detected, and IL‐27Rα^+^ spleen cells adoptive transfer was performed. STAT1/3/5 phosphorylation, proliferation and apoptosis were investigated in mixed lymphocyte reaction (MLR) with recombinant IL‐27 (rIL‐27) stimulation. Finally, IFN‐γ/ IL‐10 in graft/serum from model mice was detected. Results showed higher IL‐27Rα/IL‐27 expression in allografted group compared that syngrafted group on day 10 (top point of allorejection). IL‐27Rα^+^ spleen cells accelerated allograft rejection in vivo. rIL‐27 significantly promoted proliferation, inhibited apoptosis and increased STAT1/3/5 phosphorylation of alloreactive splenocytes, and these effects of rIL‐27 could be almost totally blocked by JAK/ STAT inhibitor and anti‐IL‐27 p28 Ab. Finally, higher IL‐27Rα^+^IFN‐γ^+^ cells and lower IL‐27Rα^+^IL‐10^+^ cells within allografts, and high IFN‐γ/low IL‐10 in serum of allorejecting mice were detected. In conclusion, these data suggested that IL‐27Rα^+^ cells apparently promoted allograft rejection through enhancing alloreactive proliferation, inhibiting apoptosis and up‐regulating IFN‐γ via enhancing STAT pathway. Blocking IL‐27 pathway may favour to prevent allorejection, and IL‐27Rα may be as a high selective molecule for targeting diagnosis and therapy for allotransplantation rejection.

## 1 INTRODUCTION

Organ transplantation is the most effective therapy for end‐stage organ failure, while accompanied acute allorejection was reported to be the leading causes of graft dysfunction.^1^, ^2^ Since long‐term immunosuppression increased the occurrence of opportunistic infections and the incidence of cancer,^3^ searching for new target molecules and illustrating related pathway is urgently needed for further understanding rejection mechanisms and modulating clinic administration.

Interleukin‐27 receptor (IL‐27R) is composed of WSX‐1 (IL‐27Rα) and gp130 (signal‐transducing subunit), expressed on CD4^+^ T cells, activated macrophages and neutrophils.^4^ And IL‐27, the ligand of IL‐27Rα, belongs to IL‐6/IL‐12 family.^5^ It was reported that engraftment of IL‐6 deficient donor into wild‐type recipient could significantly improve allograft survival through T cell lineage particularly regulatory T cells (Tregs) in non‐sensitized transplant host.^6^ IL‐27 could directly promote Th1 cell response, meanwhile inhibit the development and effector response of Th17 cells.^7^ And more importantly, data about IL‐27 displayed a controversial effect on transplantation rejection. Ayasoufi K proved that IL‐27 neutralization or receptor deficiency delayed CD8^+^ T cell recovery after murine anti‐thymocyte globulin (mATG) treatment in heart allotransplantation mouse model,^8^ and blocking IL‐27 pathway prevented from graft‐versus‐host disease (GVHD) occurrence through promoting Treg cell reconstitution and stabilizing Foxp3 expression,^9^ suggested IL‐27 with pro‐inflammation and allorejection accelerating effects. On the other hand, Yi demonstrated that IL‐27 favoured the protective effect of human placenta–derived mesenchymal stromal cells (hPMSC) in GVHD therapy by increasing CD4^+^ IL‐10^+^ IFN‐γ^+^ T cells production,^10^ up‐regulation of IL‐27 combined with rapamycin promoted IL‐10^+^CD4^+^ T cells effect in prolonging cardiac allograft survival,^11^ which suggested that IL‐27 and IL‐27Rα inhibited allorejection. So, the effect of IL‐27Rα/IL‐27 on allotransplantation rejection and its underlying mechanism was not fully understood yet.

Our previously study proved that *IL‐27Rα* up‐regulated 4 folds in allogeneic CD4^+^T cells, and more importantly, we found 10‐fold up‐regulation of *Stat1* and 4 folds increasing of *interferon gamma receptor 1 (Ifngr1)* at the same kind of CD4^+^T cells, compared with syngeneic CD4^+^T cells in Serial analysis gene expression (SAGE). Considering of the key role of CD4^+^T cells in allorejection, and the enhancing effect of IFN‐γ on allograft rejection,^12^ we postulated that IL‐27Rα, the upstream regulator of STAT1, may be the important target molecule on allograft rejection. Here, we investigated the effect of IL‐27Rα during allograft rejection, and possible signal pathway, to clarify the effect of IL‐27Rα as a pivotal molecule, on allotransplantation rejection.

## 2 MATERIALS AND METHODS

### 2.1 Animal models

Female BALB/c mice (H‐2^d^), C57BL/6 mice (H‐2^b^) and SCID Beige mice (BALB/c background) aged 6‐8 weeks and weighted 18 ± 2 g were purchased from Vital River Laboratory Animal Technology and fed in SPF condition. The skin transplantation was performed according to reference.^13^ BALB/c mice were recipients, and BALB/c and C57BL/6 were donors for syngeneic and allogeneic grafted group, respectively. The vaseline gauzes and bandage were packed on day 1 and removed on day 7 post‐transplantation. Graft was observed daily. Rejection was determined when scab area of graft was more than 50%.

Allografted SCID mice model was established according to reference.^14^ The cell adoptive transfer for allo‐SCID mice was performed after graft healed totally, then, 1.5*10^6^ sorted IL‐27Rα^+^ cells and control non‐sorted spleen cells isolated from allografted BALB/c mouse model on day 10 were adoptively transferred to SCID mouse model by intraperitoneal injection. Graft was observed daily. Rejection was determined when scab area of graft was more than 50%.

All animal studies were conducted in accordance with protocols approved by the Animal Care and Use Committee of Shandong University.

### 2.2 Spleen cell isolation, culture and reagents treatment

Spleen was isolated from mice, and single cell suspension was prepared after treated with red blood cell lysis buffer (Solarbio) according to the manufacturer's instruction. The donor cells were treated with 40 μg/mL mitomycin C (Solarbio) in 37°C, 5% CO_2_ for 30 minutes in dark and then washed three times with PBS. All cells were cultured in complete RPMI 1640 (Biological Industries) supplemented with 10% foetal bovine serum (FBS) (Biological Industries), and 100 U/mL Penicillin, and 100 µg/mL streptomycin (Hyclone) and cultured in 37°C, 5% CO_2_. The splenocytes were adjusted to a final concentration of 4 × 10^5^/well (recipient) and 2 × 10^5^/well (donor) in 96‐well and 1 × 10^6^/well (recipient) and 5 × 10^5^/well (donor) in 24‐well. Recombinant IL‐27 (rIL‐27) was dissolved in deionized water and diluted to 5, 25, 100, 400, 800 µg/mL in PBS containing 5% trehalose (DAKEWE). For the experiment of phosphorylating, alloreactive mixing lymphocyte reaction (MLR) was performed for 72 hours and replaced with new complete medium. Cells were treated with 25 ng/mL rIL‐27 for 0.25, 0.5, 1, 2 and 24 hours. For the blocking experiment, MLR was performed by allogeneic spleen cells stimulation for 72 hours and replaced with new complete medium, and then, ruxolitinib (Selleck) (0.012, 0.12 and 1.2 µmol/L) and SH‐4‐54 (Selleck) (0.15, 1.5 and 15 µmol/L) were added to cells culture for another 24 hours. Cells were incubated with 25 ng/mL rIL‐27 for 0.25 hours and then were collected to determine the phosphorylation and protein expression. ruxolitinib and SH‐4‐54 were dissolved in dimethyl sulfoxide (DMSO) and then diluted with RPMI 1640. The final concentration of anti‐IL‐27 p28 Ab (R&D) is 7.5 µg/mL (dissolved in PBS). Cell counting kit‐8 (CCK‐8) (DOJINDO) reagent was added in cell suspension for 10 µL/well, and microplate reader was used to measure OD value in 490 nm to detect cells proliferation.

### 2.3 Flow cytometry and cells sorting

Spleen cell suspension was prepared described as above, and cell number was adjusted into 1 × 10^6^ cell/100 µL PBS. For cell staining, cells were treated with 1 µg anti‐Mouse CD3e PE‐Cyanine7‐ (eBioscience), 0.25 µg anti‐Mouse CD4 FITC‐ (eBioscience) and 1 µg anti‐IL‐27Rα/PE (Bioss)‐conjugated antibody in 30 minutes in dark, respectively. For apoptosis assay, cells were suspended with 0.5 mL binding buffer and incubated with 5 µL Annexin V‐FITC and 10 µL PI (MultiSciences) for 5 minutes in dark. The cells were washed for one time and samples were acquired on a Beckman counter CytoFLEX followed by data analysis using CytExpert software. For cell sorting, cells were treated with anti‐IL‐27Rα‐PE (R&D)‐conjugated antibody (0.25 µg/10^6^ cells) for 30 minutes in dark. Wash cells for one time with sterility cold PBS buffer and sorted IL‐27Rα^+^ cells in Beckman MoFlo Astrios EQ counter.

### 2.4 H&E (haematoxylin‐eosin) staining

Graft was separated and fixed overnight, then dehydrated and embedded in paraffin blocks. The tissue sections were deparaffinized, rehydrated and stained with H&E staining kit (Servicebio). Briefly, the tissue sections were coated with haematoxylin for 5 minutes and washed with water, then covered with 1% acid ethanol regent for 5 seconds and washed with water, added blue‐promoting solution for 5 seconds, then washed with water and covered with eosin solution for 10 minutes, and dehydrated with alcohol and clearing in xylene. The image was obtained under the optical microscope.

### 2.5 Immunofluorescence and immunohistochemical staining

The graft tissue sections were deparaffinized, rehydrated and then antigen repaired with EDTA antigen repair buffer (pH 9.0) and washed three times, in 5 minutes each time with PBS (pH 7.4). For the immunofluorescent staining of CD4 (Servicebio) and IL‐27Rα (Bioss), CD4 (1:500 dilution) was represented by CY3 (red) and IL‐27Rα (1:200 dilution) was stained with FITC (green). For the immunofluorescent staining of IL‐27Rα (1:200 dilution), IL‐10 (1:100 dilution) (Bioss) and IFN‐γ (1:100 dilution) (Proteintech), the Cy5 (pink), Cy3 (red) and FITC (green) were stained. The cell nucleus was stained with DAPI (blue). The staining steps were performed according to the Servicebio immunofluorescent staining instruction, and all the second antibody and staining reagents were purchased from Servicebio. For IHC (immunohistochemical) staining, 3% hydrogen peroxide was used to block peroxidase, and anti‐IL‐27Rα (1:400 dilution) was covered overnight. The sections were incubated with second antibody and stained with DAB regents. The nuclei were stained with haematoxylin. The IOD and area count was analysed by the Image Plus Pro software. For CD4^+^ IL‐27Rα^+^cells counting, 200 CD4 positive cells were randomly selected for each section and IL‐27Rα^+^cell number was counted. For IL‐27Rα^+^IL‐10^+^/ IFN‐γ^+^ cell counting, 100 IL‐27Rα positive cell was randomly selected for each section, and the IL‐10 and IFN‐γ positive cell number were counted.

### 2.6 Western blot

The tissue was smashed and cells were washed with ice‐cold PBS and lyzed using radioimmunoprecipitation assay (RIPA) lysis buffer (Beyotime) supplemented with phenylmethanesulfonyl fluoride (PMSF, 1:100), protease inhibitor cocktail (1:100) and phosphatase inhibitor cocktail (1:50) (Beyotime) for 30 minutes on ice. The samples were centrifuged at 10 322 ×*g* for 10 minutes, and supernatant was collected to measure the concentration by BCA Protein Sample (Beyotime) treated with SDS‐PAGE Loading Buffer and then reserved in −80°C.

Protein sample was loaded into PAGE Gel (Epizyme), and performed electrophoresis, then transferred to PVDF membrane. For the total protein detection, the membranes were washed with distilled water for 5 minutes and with the stripping buffer (Servicebio) for 30 minutes. Then, washing the membranes three times with TBST buffer (Tris‐buffered saline buffer contained with Tween‐20) (Servicebio). The membranes were blocked with blocking buffer (Beyotime) for 1 hour at 25°C and incubated with the primary antibody (diluted by Primary Antibody Dilution Buffer, Beyotime) overnight. The primary antibody listed as the followings:

IL‐27 Goat pAb (1:800) (R&D); IL‐27Rα Rabbit mAb (1:2000) (R&D); p‐STAT1 (Ser727) Rabbit pAb (1:800) (Abcam); p‐STAT3 (Ser727) Rabbit pAb (1:1000) (Santa Cruz); p‐STAT5 (Tyr694) (C11C5) Rabbit mAb (1:1000) (CST); Bcl‐xL (54H6) Rabbit mAb (1:1000) (CST); Pim2 Rabbit mAb (1:800) (CST); GAPDH Rabbit pAb (1:2500) (Bioworld); β‐Actin (D165) Rabbit pAb (1:2500) (Bioworld); STAT1 Rabbit pAb (1:1500) (Proteintech); STAT3 Rabbit pAb (1:1500) (Proteintech); STAT5 Rabbit pAb (1:1500) (Proteintech).

The membranes were washed three times with TBST buffer and incubated with secondary antibody (1:10 000, dissolved to TBST) for 1 hour at room temperature, then, washed three times with washing buffer and added HRP substrate peroxide solution (Millipore). The image was obtained by Tanon 5200 imaging system scanner and analysed by ImageJ software.

### 2.7 ELISA (enzyme‐linked immunosorbent assay)

IFN‐γ, IL‐27 and IL‐10 in serum of mouse model were measured by ELISA Kit (Shanghai Enzyme‐linked Biotechnology) following the manufacture’ description. Briefly, the diluted sample was coated with conjugate reagent in the bottom of plate for 60 minutes at 37°C. The liquid was removed, and plate was washed with washing buffer for five times. Add the substrate solution for 15 minutes and then stop the colour reaction. The absorbance was measured by ELISA plate reader at OD 450 nm (Bio‐Rad). Standard curve was established to quantity the concentration of the sample.

### 2.8 Statistical analysis

Statistical analyses were performed using the GraphPad Prism 8 software and evaluated by two‐tailed *t* tests in comparisons of two groups. The survival analysis was evaluated by comparison of survival curves. *P* values < .05 were considered to be statistically significant. The quantitative values of all experiments were presented as the mean ± SD and were calculated from at least 3 independent experiments.

## 3 RESULTS

### 3.1 The dynamic expression of IL‐27Rα/IL‐27 in grafts post‐transplantation

To investigate whether IL‐27Rα/IL‐27 expression in graft was correlated with allograft rejection, we established allogeneic skin grafted mouse model and with syngeneic skin grafted mouse model as control. Grafts were isolated on 1, 7, 10, 14 and 21 day post‐transplantation, and IL‐27Rα and IL‐27 expression were detected, respectively.

**Figure 1:**
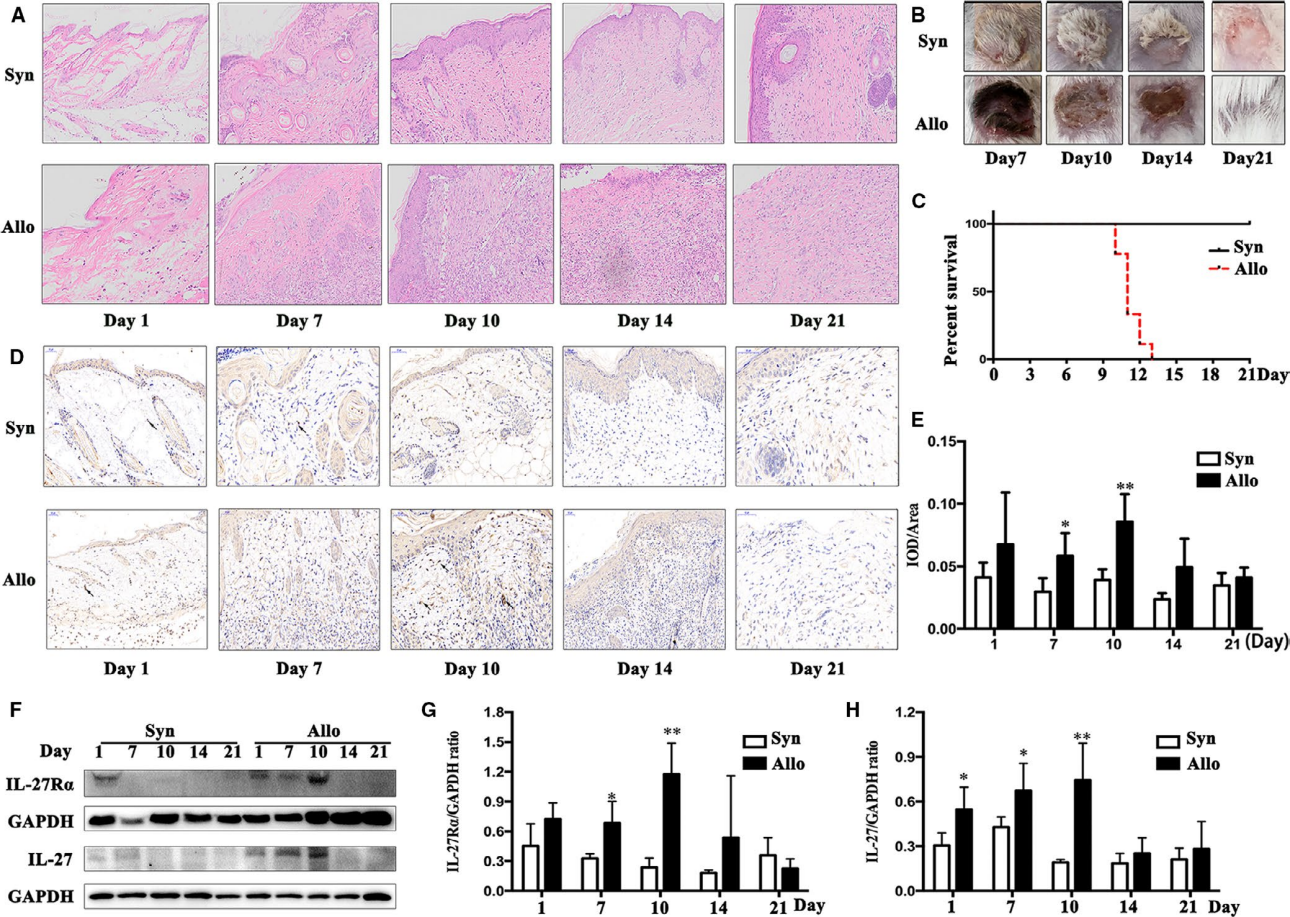
Expression of IL‐27Rα and IL‐27 in graft post‐transplantation. The graft was isolated on day 1, 7, 10, 14 and 21 post‐transplantation, respectively. The Syn and Allo group indicated syngeneic and allogeneic transplanted mouse, respectively. A, The H&E staining from day 1 to 21 post‐transplantation. B, The appearance of graft post‐transplantation. C, The survival curve of grafts. D, The IL‐27Rα expression in graft determined by immunohistochemical staining (IHC) from day 1 to 21 post‐transplantation. The arrow showed the IL‐27Rα positive cell (brown colour) in graft. E, Quantified IL‐27Rα detected with IHC by IOD/area ratio. F, The IL‐27Rα and IL‐27 p28 expression detected by Western blot. G and H, Quantified IL‐27Rα (G) and IL‐27 (H) expression in Western blot by target molecule/GAPDH ratio. *P < .05, **P < .01, ***P < .001 vs Syn group

H&E staining showed whole process of allograft rejection from day 1 to 21 post‐transplantation (Figure 1A), much more inflammatory infiltration within graft, while no inflammation observed in syngeneic grafted group. Figure 1B,C showed that allograft rejection occurred on day 10, average survival time of allograft was 11 days, no rejection was observed in syngeneic grafted group. IHC staining showed high IL‐27Rα expression on day 1 both in allograft and syngraft, the expression of IL‐27Rα was obviously increased from day 7, significantly high expressed on day 10 in allograft than syngraft (*P* < .05). On day 21, accompanied with the inflammation decreased, lower IL‐27Rα expression was detected both in two groups. IL‐27Rα positive staining mostly was detected in connective tissue and newborn blood vessels within allograft (Figure 1D). Quantified of IL‐27Rα positive staining showed higher expression of IL‐27Rα on day 7 and 10 in allograft compared with that in syngraft (Figure 1E, *P* < .05). Western blot (Figure 1F,G) further confirmed the finding in IHC staining; the highest expression of IL‐27Rα protein was also detected on day 10.

Meanwhile, the similar changes were detected in allografts for IL‐27. It was slightly increased on day 1 after transplantation both in syngraft and allograft (Figure 1F,H). On day 7 and 10, IL‐27 expression was remarkably increased in allograft compared with syngraft (*P* < .05). The highest expression of IL‐27 was found on day 10 in allograft. These results indicated that both IL‐27Rα and IL‐27 displayed highest expression on the top of rejection in allografts. And more importantly, IL‐27Rα was found in inflammatory cells‐infiltrated area, which indicated that the expression of IL‐27Rα was highly positive‐correlated with allotransplantation rejection.

### 3.2 IL‐27Rα^+^ cells promoted allograft rejection

Our previous study demonstrated IL‐27Rα^+^T cells and IL‐27Rα^+^CD68^+^ cells (macrophage) infiltrated in the graft.^15^ Considering of the pivotal role of T cells in allorejection,^16^, ^17^ we isolated graft and spleen from allogeneic and syngeneic transplantation model mice on day 10 post‐transplantation, stained with IL‐27Rα, CD3 and CD4 via immunofluorescence co‐staining and flow cytometry.

**Figure 2:**
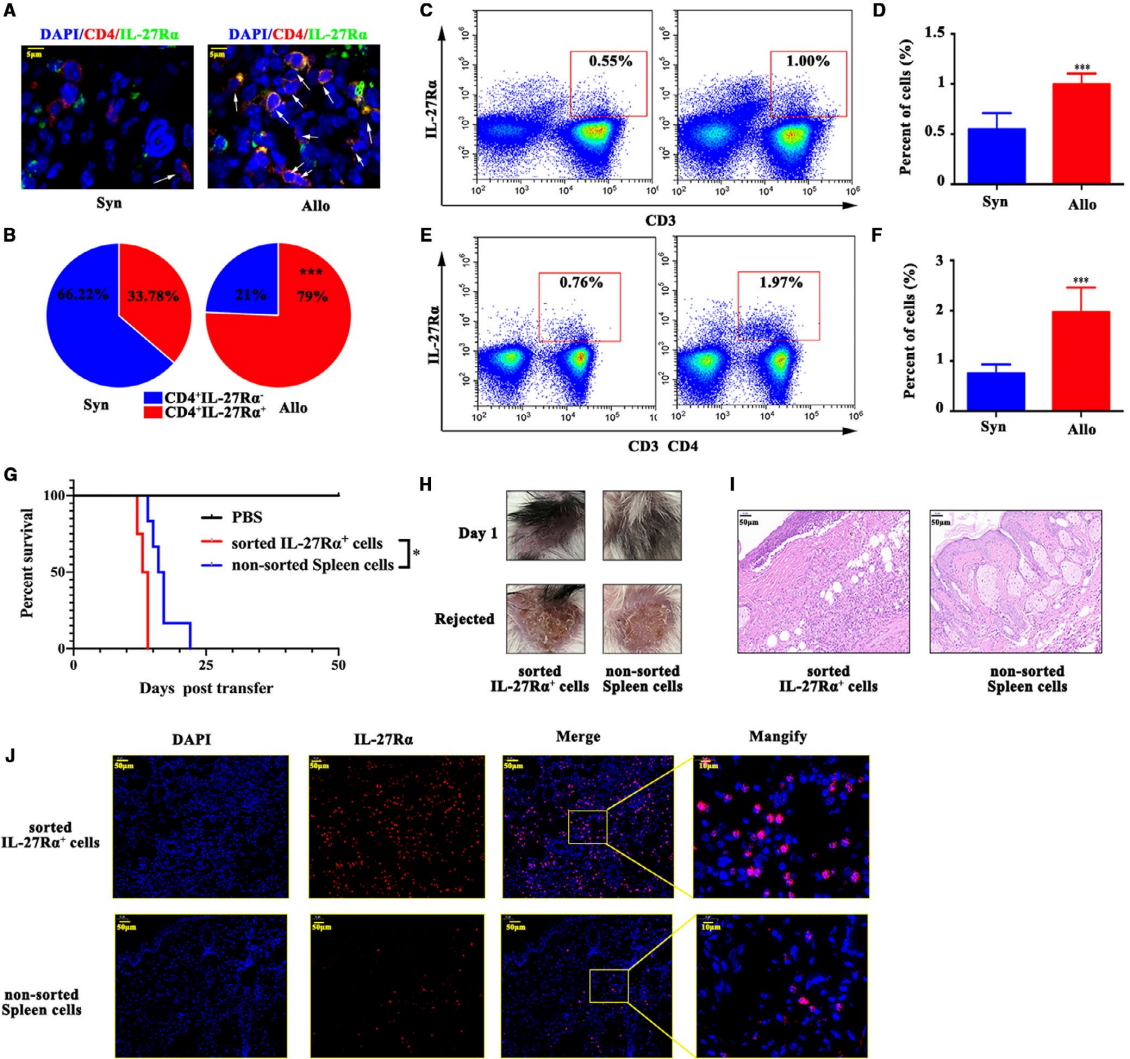
IL‐27Rα induced allograft rejection. Skin transplantation mice models were established and splenocyte and graft was separated on day 10 post‐transplantation. A, The IL‐27Rα (green) and CD4 (red) expression measured by immunofluorescent staining. The cell nucleus was stained with DAPI (blue). The arrow showed the IL‐27Rα^+^ CD4^+^cell in skin graft. B, The per cent of IL‐27Rα^+^ cell in grafted skin CD4^+^cells. C‐F, Splenic IL‐27Rα^+^ cells, CD3^+^T cells (C, D) and splenic CD4^+^T cells (E, F) detected by flow cytometry. G, The survival assay post‐cells transfer in allografted SCID mouse model. H, The appearance of allograft on day 1 post‐cells transfer and when rejection occurred. I, The H&E staining of the allograft on day 14 post‐cells transfer. J, The IL‐27Rα (green) and CD3 (red)/CD4 (red) expression measured by immunofluorescent staining. The cell nucleus was stained with DAPI (blue). *P < .05, ***P < .001

Immunofluorescence co‐staining results showed as Figure 2A,B, IL‐27Rα^+^CD4^+^ double positive cells within allograft apparently increased compared with that in syngraft (79% vs 33.78%, *P* < .05). And we also found that the percentage of IL‐27Rα^+^CD3^+^ T cells (Figure 2C,D) and IL‐27Rα^+^CD4^+^cells in CD3^+^T cells (Figure 2E,F) were both significantly increased in spleen from allografted group compared with that from syngeneic grafted group (*P* < .05). These data suggested that high expressed IL‐27Rα in allograft and in recipient's spleen tightly positively correlated with CD3^+^T cells and CD4^+^T cells, and up‐regulated IL‐27Rα in these cells was positively correlated with allorejection.

To further confirm the directive role of IL‐27Rα^+^ cells on allograft rejection, we sorted IL‐27Rα^+^ spleen cells from allografted BALB/c model on day 10 post‐transplantation and adoptively transferred to allografted SCID mouse model. Same amount of non‐sorted spleen cells from same model was adoptively transferred to allografted SCID mouse model as the control. SCID Beige mice showed impaired T and B cell development and defective natural killer (NK) cell, but normal macrophage effect.^18^

**Figure 3:**
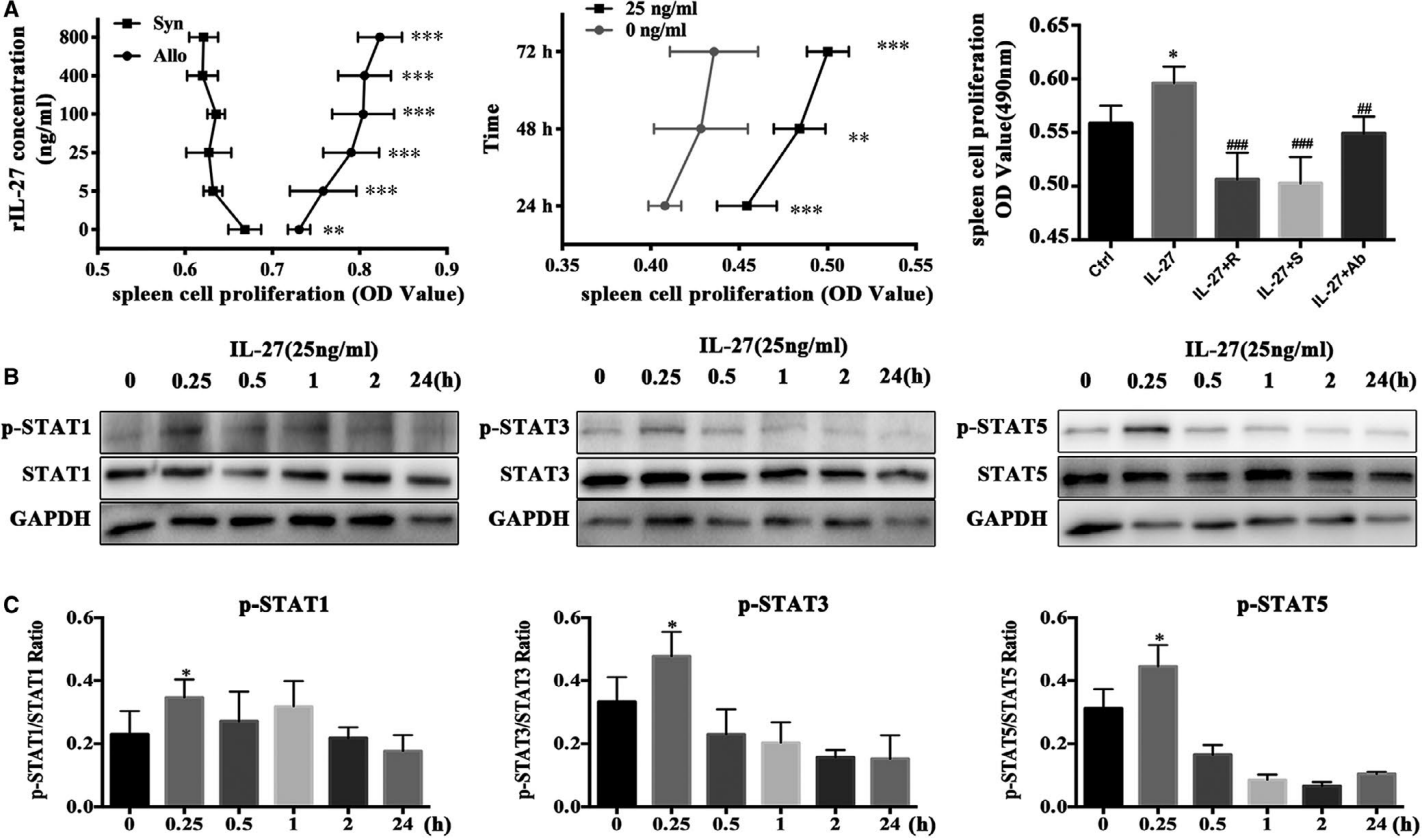
IL‐27 promoted the alloreactive spleen cells proliferation through STAT1, STAT3 and STAT5 activation. MLR were performed by syngeneic and allogeneic stimulation for 72 h. ruxolitinib (JAK inhibitor) and SH‐4‐54 (STAT inhibitor) were used to inhibit JAK/STAT activation. IL‐27 group treated with rIL‐27; IL‐27 + R group treated with rIL‐27 plus ruxolitinib; IL‐27 + S group treated with rIL‐27 plus SH‐4‐54; IL‐27 + Ab group treated with rIL‐27 and anti‐IL‐27 p28. A, First, cultured mixture splenic cells treated with different concentration of rIL‐27 for 72 h. Second, 25 ng/mL rIL‐27 was added to cultured mixture splenic cells for 24, 48 and 72 h. The third, cultured mixture splenic cells treated with rIL‐27 (25 ng/mL), rIL‐27 + ruxolitinib (0.12 µmol/L) and rIL‐27 + SH‐4‐54 (1.5 µmol/L), respectively for 72 h. Finally, CCK8 was added to wells, continue to culture for another 2 h and OD value (490 nm) was measured. B and C, The phosphorylation and total protein expressions of STAT1/3/5 pathway in different times for mixture splenic cells detected by Western blot (B) and analyzed by ImageJ (C). *P < .05, **P < .01, ***P < .001 vs Control group. ^#^ *P* < .05, ^##^ *P* < .01, ^###^ *P* < .001 vs IL‐27 group

Figure 2G clearly showed that no graft rejection happened in allografted SCID mouse model. After IL‐27Rα^+^ spleen cells adoptive transfer, the rejection of allograft occurred earlier than non‐sorted spleen cells (survival time, 13.5 days vs 16.5 days, *P* < .05). The allograft scabbed when allograft rejected (Figure 2H). On day 14 post‐cells transfer, IL‐27Rα^+^ cells group showed much more severe inflammation and much more IL‐27Rα^+^ cells infiltration than that in non‐sorted spleen cells transfer group (Figure 3I,J). These results indicated that IL‐27Rα^+^ cells apparently promoted allograft rejection.

### 3.3 rIL‐27 in vitro enhanced proliferation of alloreactive splenocytes via activating STAT1/3/5

Since the IL‐27Rα^+^ cells from spleen could promote allograft rejection, therefore, we further investigated the effective mechanism of IL‐27Rα/IL‐27 in vitro with mixed lymphocyte reaction (MLR) under stimulation of rIL‐27. Figure 3A showed that rIL‐27 obviously promoted alloreactive cell proliferation in all checked concentration (5, 25, 100, 400 and 800 ng/mL, *P* < .05), and even lower concentration at 25 ng/mL could apparently promote cells proliferation at all checked point (24, 48 and 72 hours) (*P* < .05). And we found the effect on proliferation of rIL‐27 could be obviously down‐regulated when added anti‐IL‐27p28 antibody into system (*P* < .05).

It has been reported that IL‐27 signalled through JAK/STAT axis^19^, ^20^ and STAT1, STAT3 and STAT5 were all up‐regulated during allorejection.^14^, ^21^, ^22^, ^23^ To further understand whether IL‐27 in MLR was also signalled through STAT1, STAT3 and STAT5 phosphorylation, we added rIL‐27 (25 ng/mL) into MLR system, cultured for 0.25, 0.5, 1, 2 and 24 hours. The results showed as Figure 3B,C, rIL‐27 obviously activated STAT1, STAT3 and STAT5 phosphorylation, and all peaked at 0.25 hours, especially p‐STAT3 and p‐STAT5.

**Figure 4:**
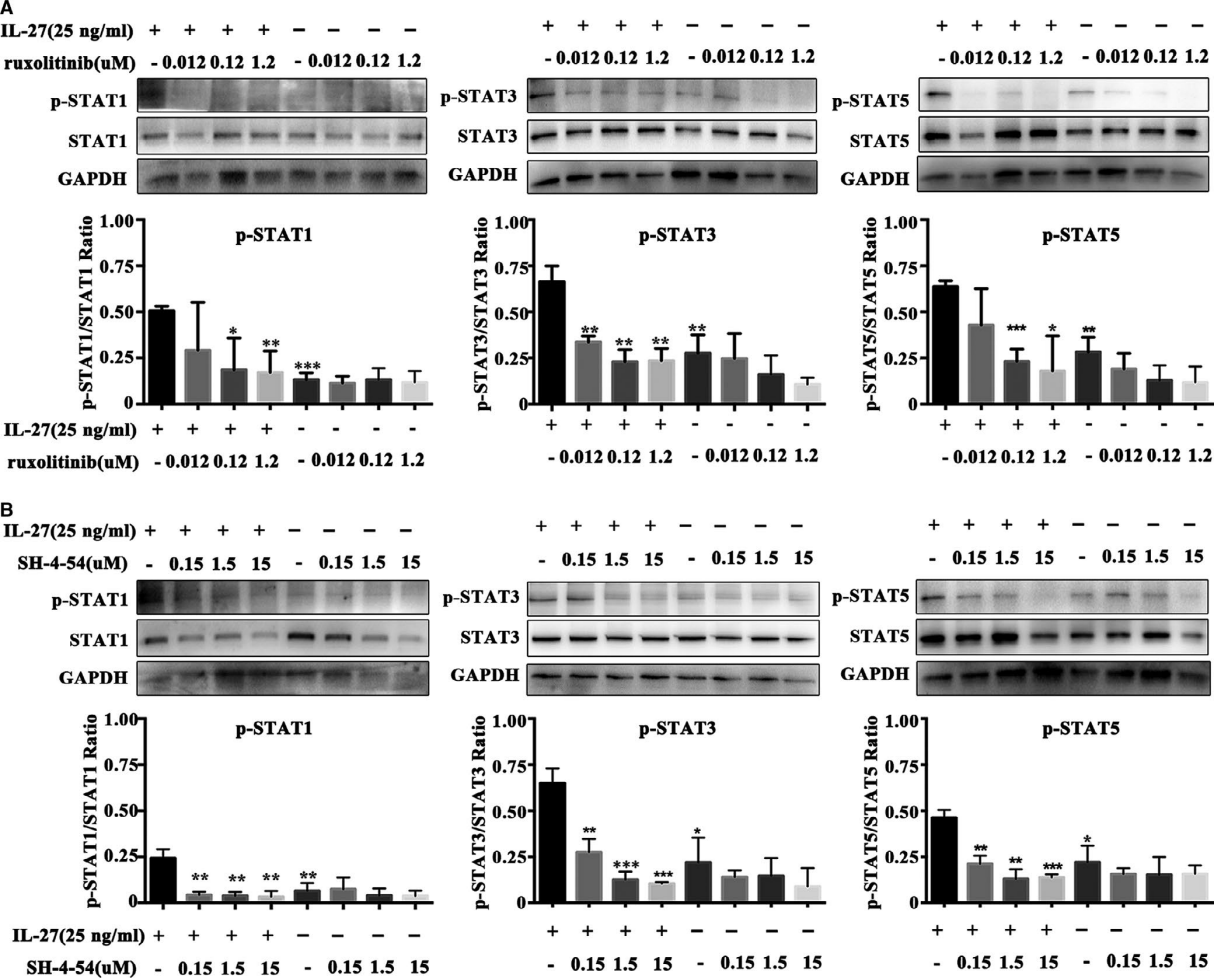
JAK/STAT inhibitor blocking IL‐27 effect on STAT1, STAT3 and STAT5 phosphorylation. MLR was performed by allogeneic stimulation for 72 h, then treated with ruxolitinib (0.12 µmol/L) and SH‐4‐54 (1.5 µmol/L) for 24 h, and finally stimulated with rIL‐27(25 ng/mL) for 15 min. IL‐27 group treated with rIL‐27; IL‐27 + R group treated with rIL‐27 and ruxolitinib; IL‐27 + S group treated with rIL‐27 along with SH‐4‐54; IL‐27 + Ab group treated with rIL‐27 and anti‐IL‐27 p28. A, The inhibition of STAT pathway by different concentration of ruxolitinib. B, The inhibition of STAT pathway by different concentration of SH‐4‐54. Band intensities was measured ImageJ. Phosphorylation indicated by the ratio of phosphorylation value to the total protein value. *P < .05, **P < .01, ***P < .001 vs Control group

Furthermore, we repeated above experiment with rIL‐27 (25 ng/mL) stimulation pre‐treated with ruxolitinib (JAK1/2 inhibitor, 0.012, 0.12 and 1.2 µmol/L) and SH‐4‐54 (STAT inhibitor, 0.15 1.5 and 15 µmol/L). The results showed that p‐STAT1, p‐STAT3 and p‐STAT5 of alloreactive cells obviously decreased compared with rIL‐27 stimulation alone (Figure 4A, *P* < .05). And interestingly, we found that 0.12 and 1.2 µmol/L ruxolitinib and all checked concentration of SH‐4‐54 could apparently inhibit higher expression of p‐STAT1, p‐STAT3 and p‐STAT5 stimulated by rIL‐27 (Figure 4B, *P* < .05), which further confirmed that effect of IL‐27Rα activation in MLR was through STAT signal pathway.

### 3.4 rIL‐27 inhibited apoptosis of alloreactive cells via enhancing STAT1/3/5

**Figure 5:**
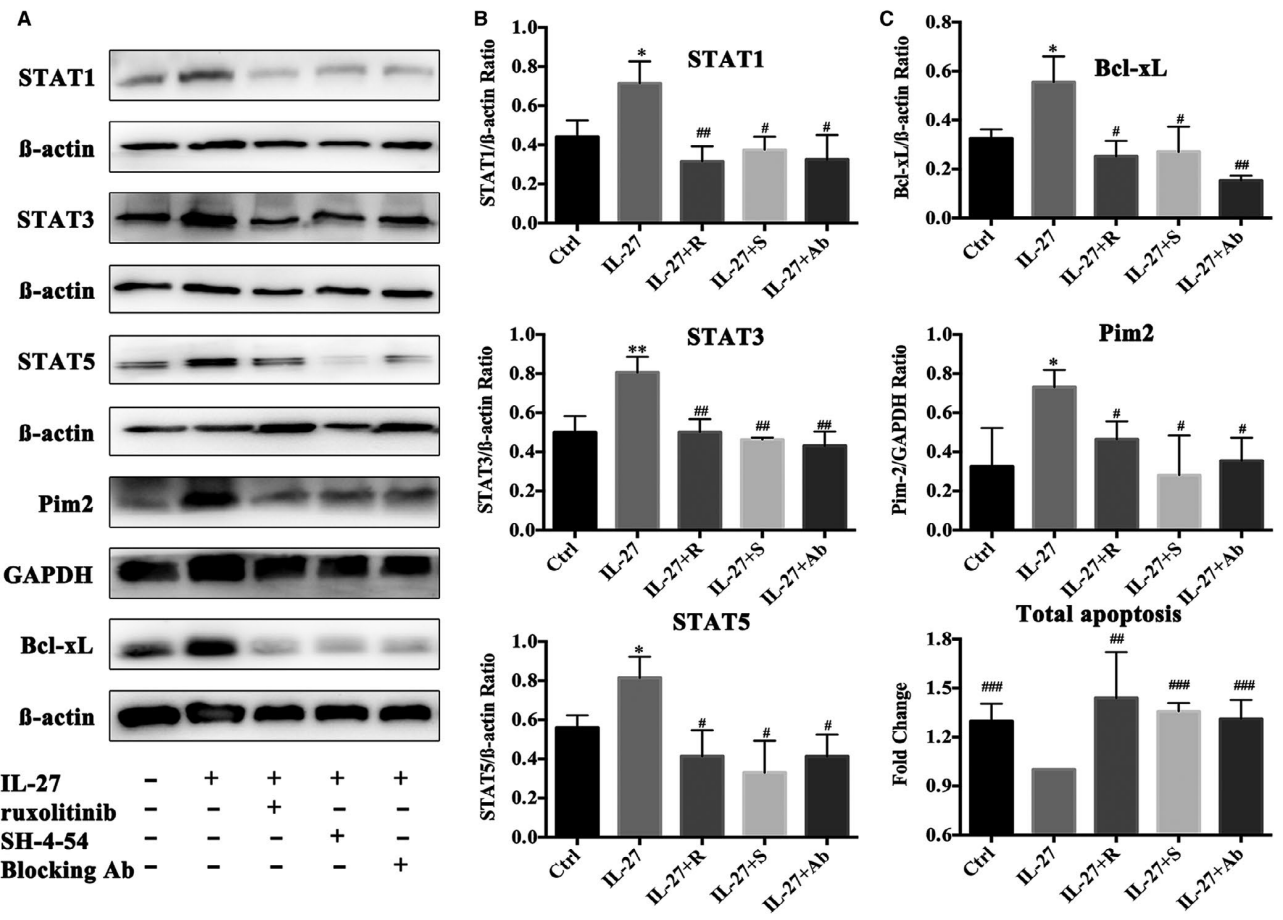
The up‐regulation STAT pathway by rIL‐27 resulted in anti‐apoptosis of alloreactive spleen cells. Alloreactive MLR were performed with treated by rIL‐27, ruxolitinib, SH‐4‐54 and anti‐IL‐27 p28 Ab for 72 h. IL‐27 group treated with rIL‐27. IL‐27 + R group treated with rIL‐27 and ruxolitinib. IL‐27 + S group treated with rIL‐27 along with SH‐4‐54. IL‐27 + Ab group treated with rIL‐27 and anti‐IL‐27 p28. A, STAT1, STAT3, STAT5, Bcl‐xL and Pim2 expression were detected by Western blot. B, Band intensities in Western blot for STAT1, STAT3, STAT5 expression were measured ImageJ. C, The band intensities in Western blot for Bcl‐xL and Pim2 expression were measured ImageJ. D, Alloreactive spleen cell apoptosis detected by flow cytometry. Fold change means the ratio of apoptotic cells in each group to those in IL‐27 group. *P < .05, **P < .01, ***P < .001 vs Control group. ^#^ *P* < .05, ^##^ *P* < .01, ^###^ *P* < .001 vs IL‐27 group

Our previously data indicated that apoptosis of alloreactive cells played an important role in allorejection (Data not shown). According to report,^24^ the downstream of STAT pathway includes cells proliferation and apoptosis. Considering that we already proved that proliferation increased by rIL‐27 stimulation was through STAT pathway, further we investigated whether cell apoptosis was also affected through STAT pathway under rIL‐27 stimulation. The results were shown as Figure 5A,B, and the expression of STAT1, STAT3 and STAT5 all apparently increased under rIL‐27 stimulation for 72 hours (25 ng/mL) (*P* < .05, vs Control). JAK inhibitor (ruxolitinib), STAT inhibitor (SH‐4‐54) and IL‐27 antibody blocking (anti‐IL‐27 p28) all could inhibit the expression of STAT1/3/5 (*P* < .05, vs rIL‐27 alone group), and no difference was detected compared with Control group.

Figure 5A,C showed that anti‐apoptosis protein Bcl‐xL and Pim2, the molecules in downstream of JAK/STAT,^25^, ^26^, ^27^ were both obviously up‐regulated under rIL‐27 stimulation (vs Control group, *P* < .05). And more importantly, we found that ruxolitinib (0.12 µmol/L) and SH‐4‐54 (1.5 µmol/L) apparently down‐regulated high expression of Bcl‐xL and Pim2 induced by rIL‐27 (vs IL‐27 group, *P* < .05). Results of flow cytometry proved that rIL‐27 apparently inhibited total apoptosis of alloreactive cells, and STAT pathway inhibition obviously promoted apoptosis (Figure 5D, vs IL‐27 group, *P* < .05). Interestingly, we found that neutralization with IL‐27 p28 (the main active fragment of IL‐27) antibody significantly inhibited the expression of Bcl‐xL and Pim2 and promoted total apoptosis (Figure 5A,C, vs IL‐27 group, *P* < .05), which suggested that IL‐27 p28 may dominate to inhibit apoptosis via increasing the expression of STAT1, STAT3 and STAT5.

### 3.5 IL‐27Rα activation was prone to higher IFN‐γ and lower IL‐10 expression on allorejection

Considering that pro‐inflammatory/immunosuppressive cytokine both participated in alloreactive cells mediated rejection,^28^ we detected IL‐27Rα, IFN‐γ (pro‐inflammatory cytokine) and IL‐10 (immunosuppressive cytokine) within graft through immunofluorescent co‐staining on day 10 post‐transplantation, to investigate IL‐27 signalling pathway in allografted mice models.

**Figure 6:**
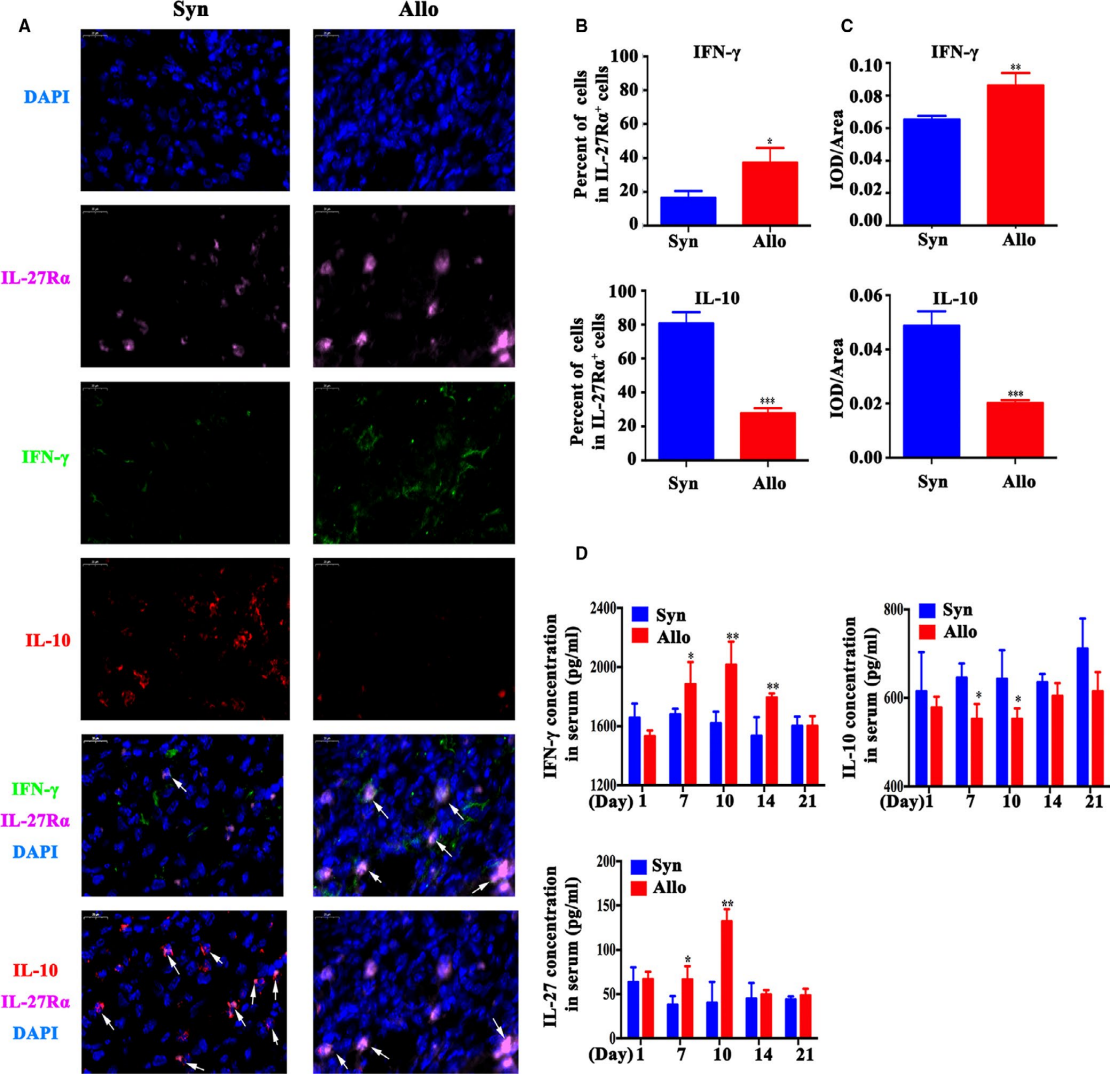
IFN‐γ, IL‐10 and IL‐27 expression assay. Syngeneic and allogeneic skin mouse model was established and was represented by Syn and Allo group. IFN‐γ, IL‐10 and IL‐27 level were measured in graft and serum. A, The IFN‐γ (green), IL‐10 (red) and IL‐27Rα (pink) expression of graft was measured by immunofluorescent staining on day 10. The cell nucleus was stained with DAPI (blue). Scale bar = 20 μm. The arrow showed the IFN‐γ^+^/ IL‐10^+^ and IL‐27 Rα ^+^cells. B, The per cent of IFN‐γ or IL‐10 positive cell in IL‐27Rα positive cells on day 10. C, The IFN‐γ or IL‐10 expression was quantified by IOD/Area value. D, The level of IFN‐γ, IL‐10 and IL‐27 in serum was measured by ELISA from day 1 to day 21 post‐transplantation. *P < .05, **P < .01, ***P < .001 vs Syn group

Figure 6A revealed the IL‐27Rα^+^ cells were up‐regulation in allograft with higher IFN‐γ and lower IL‐10 expression simultaneously on day 10 post‐transplantation. Compared with syngraft, the proportion of IL‐27Rα^+^IL‐10^+^cells in allograft had a markedly reduction, while IL‐27Rα^+^IFN‐γ^+^ cells obviously increased (*P* < .05, Figure 6B). These results indicated that IFN‐γ high expression and IL‐10 low expression was affected on allorejection. Quantified with IOD/area value in Figure 6C further proved this result.

To understand the systemic effect of IL‐27Rα high expression on allograft rejection, dynamic level of IFN‐γ and IL‐10 in serum of grafted mouse model post‐transplantation were measured by ELISA. The results showed that during transplantation rejection, the level of these cytokine changed depended on graft condition. Compared with the syngeneic grafted group, the allografted group showed much higher IFN‐γ and lower IL‐10 on day 10 (*P* < .05, Figure 6D), which suggested that IL‐27Rα high expression in grafts was positively correlated with higher pro‐inflammatory and lower immunosuppressive cytokine in serum.

To investigate whether IL‐27 in peripheral blood was also changed during allotransplantation rejection, IL‐27 in model mice serum was also detected with ELISA, and the results showed that IL‐27 also apparently increased on the day 10 after allogeneic transplantation (*P* < .05, vs syngeneic grafted group, Figure 6D), which confirmed that higher IL‐27 was consisted with high expression of IL‐27Rα and IL‐27 within allograft when rejection occurred. Taken together, these results revealed that the IL‐27Rα activation promoted much more IFN‐γ and less IL‐10 releasing, and these cytokine may be as an effective factor through STAT pathway to increase alloreactive cells proliferation, inhibit apoptosis, and to promote allografts allorejection.

## 4 DISCUSSION

The role of IL‐27Rα/IL‐27 pathway in acute allograft rejection have not been reported until now. In this study, we demonstrated the IL‐27Rα activation could promote allorejection through STAT pathway both in vivo and in vitro. We proved that IL‐27Rα was up‐regulation in allograft when rejection occurred. IL‐27Rα^+^ cells could promote allograft rejection in vivo. And the effector cells mainly belonged to T cells and CD4^+^T cell. In vitro experiments demonstrated that IL‐27Rα activation could up‐regulate alloreactive cells proliferation and inhibit apoptosis. And more importantly, we found that increased IFN‐γ, IL‐27 and decreased IL‐10 both in rejecting allograft and in serum of allografted mice may be as the effective cytokines after STAT pathway activation through IL‐27Rα. Our funding may benefit for IL‐27Rα targeted study and may supply a new strategy for diagnosis and treatment of allotransplantation rejection.

Acute rejection reflected pro‐inflammatory response and directly modulated or generated allograft failure in clinic.^29^ Our previously study proved that up‐regulated expression of IL‐27Rα, STAT1 and down‐regulated of Pim2 in allotransplantation, which all belongs to IL‐27Rα‐STAT pathway, could prolong allograft survival.^14^, ^27^ Here, we focused on IL‐27Rα‐STAT pathway to search for the new target for modulation of allograft rejection.

It was reported that IL‐27 binding its receptor IL‐27Rα induced the heterogeneous JAK/STAT signalling cascade such as STAT1, STAT3 and STAT5 phosphorylation in T cells.^30^ IL‐27 signalling may result in inhibitory or stimulatory inflammation reaction by modulating T cell response and controlling the cytokine secretion according to condition. Cui proved IL‐27 signalling displayed anti‐inflammatory function on epithelial cells to decrease IL‐6, TNF‐α, GM‐CSF and CXCL1 expression.^31^ In allergic airway inflammation, IL‐27 favoured Foxp3^+^ Treg function to inhibit T cell proliferation and finally attenuated inflammatory.^32^ IL‐27 pathway promoted activated T cells to switch to Th2 polarization and up‐regulate IL‐10 secretion of CD4^+^T cells.^33^ However, in contrast, IL‐27 exhibits pro‐inflammation property in antiviral immunity and T cell response. In influenza infection, IL‐27 augmented NK cells anti‐virus responses and IFN‐γ secretion via the NKG2D receptor.^34^ IL‐27 also strongly stimulated CD4^+^T cell proliferation and Th1 cytokine production by up‐regulating the antigen‐processing capability of DC in the Staphylococcus aureus infection.^35^ Considering that severe CD4^+^T cell infiltration in graft and its key role in allorejection,^17^ and the up‐regulation of IL‐27Rα and IFN‐γ in allogeneic CD4^+^T cells reported in our previously report along with others exhibited that IFN‐γ‐producing CD4^+^T cell predominated allograft rejection response,^36^, ^37^ here we investigated the effect of IL‐27Rα and related pathway in allograft rejection, mainly focused on T cells.

To understand the relationship between IL‐27Rα pathway and allorejection, we established syngeneic and allogeneic transplantation mouse model and detected expression of IL‐27Rα and IL‐27 in graft and in recipient's spleen during transplantation. The graft appearance observation and H&E staining indicated the rejection progress. The inflammation infiltration was started from day 1, and severe inflammation response occurred on day 10 post‐transplantation. Importantly, we noted the highest expression of IL‐27Rα and IL‐27 on day 10, which was the top period of allorejection (tolerance condition was observed in syngeneic transplantation group), indicated that these inflammation‐infiltrated cells may response to the higher IL‐27Rα modulation. Then, rejecting allograft was found up‐regulated IL‐27Rα expression in CD4^+^ T cells within graft and spleen. Our previous work has proved that IL‐27Rα expressed on the infiltrated T cell and macrophage of the allograft^15^; therefore, we sorted IL‐27Rα^+^ cells from the spleen to prove the role of IL‐27Rα^+^ effector cells in allografted SCID mouse model. Interesting, we found these cells induced allograft rejection and occurred rejection earlier than non‐sorted spleen cells. The rejected allograft showed severe inflammation cells. These results proved the IL‐27Rα^+^ cells could infiltrate the allograft and induced allograft rejection.

To investigate the underlying mechanism of IL‐27Rα activation on allorejection, we performed MLR with or without rIL‐27 stimulation. We found that rIL‐27 could promote alloreactive cell proliferation, which dominantly depended on enhancing STAT1/3/5 phosphorylation. rIL‐27 treatment resulted in the up‐regulation of STAT1/3/5 expression and cascaded with increasing proliferation and inhibited apoptosis along with higher expression of anti‐apoptosis protein Bcl‐xL and Pim2. With the JAK‐STAT inhibitor and neutralization antibody treatment, further confirmed lower apoptosis and high proliferation all through STAT pathway. Interestingly, initial reports revealed that IL‐35 (p35/Ebi3), which shared the same subunit Ebi3 with IL‐27 (p28/Ebi3), could suppress T cell proliferation, converse into suppressive iTr35 cells.^38^, ^39^ Meanwhile, recent evidences indicated IL‐35 promoted Treg but not Th1 differentiation, whereas IL‐27 (Ebi3 and p28) exhibited more Th1 differentiation and less Treg differentiation. Furthermore, Ebi3 alone did not induce T help cell differentiation.^40^ So, the effect of IL‐27 p28 and Ebi3 may show different effect. In our study, rIL‐27 was composed of IL‐27 p28 and Ebi3. Interestingly, we found that neutralization of IL‐27 p28 exhibited the similar effect as that of inhibitor of STAT pathway with ruxolitinib and SH‐4‐54. These findings suggested that IL‐27 mainly depended on p28 subunit to increase proliferation and inhibit apoptosis via activating STAT pathway.

Considering of the fact that up‐regulating IL‐27Rα and IL‐27 was in inflammatory disease and rejecting allograft, we postulated that IL‐27Rα pathway may promote allorejection via STAT pathway through pro‐inflammatory or immunosuppressive cytokine. IL‐10 was regarded as an inflammatory inhibitory cytokine, which prolonging the survival of skin allograft and increasing the proportion of Treg cells.^41^ IFN‐γ was classical pro‐inflammatory cytokine. We performed IL‐27Rα/ IL‐10/ IFN‐γ staining for allograft on top period of allorejection. Results indicated that IL‐10^+^ IL‐27Rα^+^ cells strongly decreased, while the IFN‐γ^+^ IL‐27Rα^+^ cells increased in allograft. At the same time, increased IFN‐γ and decreased IL‐10 in serum of allografted model mice were detected. Since that IFN‐γ predicted acute rejection and IL‐10 represented allograft tolerance,^42^ these results indicated the local graft and systemic change in allogeneic transplanted mouse exhibited severe inflammatory response, IL‐27Rα^+^ cell took part in pro‐inflammation response. IL‐27Rα pathway prone to much more IFN‐γ and less IL‐10 release to accelerate allorejection.

Recently, it was reported that IL‐2Rα/IL‐27 pathway displayed contrast effect in allogeneic transplantation rejection In humanized xenogeneic GVHD NOD/SCID model, IL‐27 favoured human placenta–derived MSCs to promote generation of CD4^+^IL‐10^+^IFN‐γ^+^ T cells and alleviate GVHD symptoms.^10^ By enhancing IL‐10/ IFN‐γ expression ratio in the alloantigen specific CD4^+^ T cells, IL‐27 adenovirus associated virus (AAV) transduction promoted the regulation of rapamycin to prolong the allograft survival.^11^ In contrast, our present study showed that IL‐27Rα was up‐regulated in allogeneic CD4^+^T cells. Recently report revealed that anti‐IL‐27 p28 antibody treatment could magnify the protection capability of TLR7 ligand R848 to GVHD by strongly activating regulatory T cell.^43^ In our study, we found that anti‐IL‐27 p28 could almost totally neutralize that effect of rIL‐27 on proliferation and apoptosis via STAT pathway.

In a summary, our data suggested that high expressed IL‐27Rα in allogeneic graft promoted allorejection through enhancing proliferation and anti‐apoptosis, via activating STAT pathway. The effective route may be mainly through producing much more pro‐inflammatory cytokine IFN‐γ and less immunosuppressive cytokine IL‐10 in graft and in peripheral blood. Blocking IL‐27 pathway may favour to prevent allorejection, and IL‐27Rα may be as a high selective molecule for targeting diagnosis and therapy for allotransplantation rejection.

## CONFLICTS OF INTEREST

The authors have no conflicts of interest to disclose.

## AUTHOR CONTRIBUTION

**Shanshan Zhao:** Conceptualization (equal); Data curation (lead); Formal analysis (lead); Investigation (lead); Methodology (lead); Validation (lead); Visualization (lead); Writing‐original draft (equal); Writing‐review & editing (equal). **Ting Liang:** Methodology (supporting); Project administration (supporting); Resources (supporting); Supervision (supporting); Visualization (supporting). **Chao Zhang:** Funding acquisition (supporting); Methodology (supporting); Project administration (supporting); Resources (supporting); Supervision (supporting). **Dai Shi:** Investigation (supporting); Methodology (supporting); Validation (supporting). **Wen Jiang:** Investigation (supporting); Methodology (supporting); Validation (supporting). **Chen Su:** Investigation (supporting); Methodology (supporting); Validation (supporting). **Guihua Hou:** Conceptualization (lead); Funding acquisition (lead); Methodology (supporting); Project administration (lead); Supervision (lead); Visualization (equal); Writing‐original draft (equal); Writing‐review & editing (equal).

## Data Availability

The data that support the findings of this study are available from the corresponding author upon request.

## References

[1] Cui Y , Liu K , Monzon‐Medina ME , et al. Therapeutic lymphangiogenesis ameliorates established acute lung allograft rejection. J Clin Invest. 2015;125:4255‐4268. 26485284 10.1172/JCI79693 PMC4639995

[2] Bouquegneau A , Loheac C , Aubert O , et al. Complement‐activating donor‐specific anti‐HLA antibodies and solid organ transplant survival: a systematic review and meta‐analysis. PLoS Med. 2018;15(5):e1002572. 29799874 10.1371/journal.pmed.1002572 PMC5969739

[3] Sachs DH . Immune tolerance, xenografts, and large‐animal studies in transplantation. Ann Am Thorac Soc. 2017;14:S220‐S225. 28945472 10.1513/AnnalsATS.201607-534MG PMC5711340

[4] Iwasaki Y , Fujio K , Okamura T , Yamamoto K . Interleukin‐27 in T cell immunity. Int J Mol Sci. 2015;16:2851‐2863. 25633106 10.3390/ijms16022851 PMC4346869

[5] Fabbi M , Carbotti G , Ferrini S . Dual roles of IL‐27 in cancer biology and immunotherapy. Mediators Inflamm. 2017;2017:3958069. 28255204 10.1155/2017/3958069 PMC5309407

[6] Ge F , Yuan S , Su L , et al. Alteration of innate immunity by donor IL‐6 deficiency in a presensitized heart transplant model. PLoS One. 2013;8:e77559. 24147024 10.1371/journal.pone.0077559 PMC3797753

[7] Qiu S‐L , Duan M‐C , Liang Y , et al. Cigarette smoke induction of interleukin‐27/WSX‐1 regulates the differentiation of Th1 and Th17 cells in a smoking mouse model of emphysema. Front Immunol. 2016;7:553. 27994590 10.3389/fimmu.2016.00553 PMC5136545

[8] Ayasoufi K , Zwick DB , Fan R , et al. Interleukin‐27 promotes CD8+ T cell reconstitution following antibody‐mediated lymphoablation. JCI Insight. 2019;4:e125489. 10.1172/jci.insight.125489 PMC6483639 30944247

[9] Belle L , Agle K , Zhou V , et al. Blockade of interleukin‐27 signaling reduces GVHD in mice by augmenting Treg reconstitution and stabilizing Foxp3 expression. Blood. 2016;128:2068‐2082. 27488350 10.1182/blood-2016-02-698241 PMC5073185

[10] Yi J , Chen Z , Xu F , et al. IL‐27 promotes human placenta‐derived mesenchymal stromal cell ability to induce the generation of CD4(+)IL‐10(+)IFN‐γ(+) T cells via the JAK/STAT pathway in the treatment of experimental graft‐versus‐host disease. J Immunol. 2019;202:1124‐1136. 30651340 10.4049/jimmunol.1800963 PMC6360257

[11] Le Texier L , Thebault P , Carvalho‐Gaspar M , et al. Immunoregulatory function of IL‐27 and TGF‐β1 in cardiac allograft transplantation. Transplantation. 2012;94:226‐233. 22790384 10.1097/TP.0b013e31825b0c38 PMC3442234

[12] Zhou J , Qin L , Yi T , et al. Interferon‐γ‐mediated allograft rejection exacerbates cardiovascular disease of hyperlipidemic murine transplant recipients. Circ Res. 2015;117:943‐955. 26399469 10.1161/CIRCRESAHA.115.306932 PMC4636943

[13] Hao J , Zhang C , Liang T , Song J , Hou G . rFliC prolongs allograft survival in association with the activation of recipient Tregs in a TLR5‐dependent manner. Cell Mol Immunol. 2014;11:206‐214. 24097035 10.1038/cmi.2013.44 PMC4003372

[14] Xu J , Wang D , Zhang C , et al. Alternatively expressed genes identified in the CD4+ T cells of allograft rejection mice. Cell Transplant. 2011;20:333‐350. 21294963 10.3727/096368910X552844

[15] Zhao S , Shi D , Su C , et al. IL‐27Rα: a novel molecular imaging marker for allograft rejection. Int J Mol Sci. 2020;21:E1315. 32075272 10.3390/ijms21041315 PMC7072931

[16] Kwok C , Pavlosky A , Lian D , et al. Necroptosis is involved in CD4+ T cell‐mediated microvascular endothelial cell death and chronic cardiac allograft rejection. Transplantation. 2016;101:1. 10.1097/TP.0000000000001578 29633982

[17] Salcido‐Ochoa F , Hue SS‐S , Peng S , et al. Histopathological analysis of infiltrating T cell subsets in acute T cell‐mediated rejection in the kidney transplant. World J Transplant. 2017;7:222‐234. 28900605 10.5500/wjt.v7.i4.222 PMC5573898

[18] SCID‐bg mice as xenograft recipients. Lab Anim. 1997;31:163‐168. 9175014 10.1258/002367797780600107

[19] Jung J‐Y , Gleave Parson M , Kraft JD , et al. Elevated interleukin‐27 levels in human neonatal macrophages regulate indoleamine dioxygenase in a STAT‐1 and STAT‐3‐dependent manner. Immunology. 2016;149:35‐47. 27238498 10.1111/imm.12625 PMC4981608

[20] Dong Z , Tai W , Lei W , Wang Y , Li Z , Zhang T . IL‐27 inhibits the TGF‐β1‐induced epithelial‐mesenchymal transition in alveolar epithelial cells. BMC Cell Biol. 2016;17:7. 26932661 10.1186/s12860-016-0084-x PMC4774182

[21] Metcalfe SM , Muthukumarana PADS . Transplantation tolerance: gene expression profiles comparing allotolerance vs. allorejection. Int Immunopharmacol. 2005;5:33‐39. 15589457 10.1016/j.intimp.2004.09.009

[22] Higazi HMKI , He L , Fang J , et al. Loss of Jak2 protects cardiac allografts from chronic rejection by attenuating Th1 response along with increased regulatory T cells. Am J Transl Res. 2019;11:624‐640. 30899367 PMC6413256

[23] Ehx G , Somja J , Warnatz H‐J , et al. Xenogeneic graft‐versus‐host disease in humanized NSG and NSG‐HLA‐A2/HHD mice. Front Immunol. 2018;9:1943. 30214443 10.3389/fimmu.2018.01943 PMC6125392

[24] Hassel JC , Winnemöller D , Schartl M , Wellbrock C . STAT5 contributes to antiapoptosis in melanoma. Melanoma Res. 2008;18:378‐385. 19011510 10.1097/CMR.0b013e32830ce7d7

[25] Liu Z , Liu H , Yuan X , et al. Downregulation of Pim‐2 induces cell cycle arrest in the G(0)/G(1) phase via the p53‐non‐dependent p21 signaling pathway. Oncol Lett. 2018;15:4079‐4086. 29541172 10.3892/ol.2018.7865 PMC5835926

[26] Bousoik E , Montazeri AH . "Do we know jack" about JAK? A closer look at JAK/STAT signaling pathway. Front Oncol. 2018;8:287. 30109213 10.3389/fonc.2018.00287 PMC6079274

[27] Liu H , Zhang C , Liang T , Song J , Hao J , Hou G . Inhibition of Pim2‐prolonged skin allograft survival through the apoptosis regulation pathway. Cell Mol Immunol. 2012;9:503‐510. 23085945 10.1038/cmi.2012.41 PMC4002220

[28] Li S , Yu J , Guo C , Jie Y , Pan Z . The balance of Th1/Th2 and LAP+Tregs/Th17 cells is crucial for graft survival in allogeneic corneal transplantation. J Ophthalmol. 2018;2018:5404989. 29576879 10.1155/2018/5404989 PMC5822769

[29] Christakoudi S , Runglall M , Mobillo P , et al. Development of a multivariable gene‐expression signature targeting T‐cell‐mediated rejection in peripheral blood of kidney transplant recipients validated in cross‐sectional and longitudinal samples. EBioMedicine. 2019;41:571‐583. 30833191 10.1016/j.ebiom.2019.01.060 PMC6441872

[30] Villarino AV , Huang E , Hunter CA . Understanding the pro‐ and anti‐inflammatory properties of IL‐27. J Immunol. 2004;173:715. 15240655 10.4049/jimmunol.173.2.715

[31] Cui B , Lu S , Lai L , et al. Protective function of interleukin 27 in colitis‐associated cancer via suppression of inflammatory cytokines in intestinal epithelial cells. Oncoimmunology. 2017;6:e1268309. 28344880 10.1080/2162402X.2016.1268309 PMC5353929

[32] Nguyen QT , Jang E , Le HT , et al. IL‐27 targets Foxp3+ Tregs to mediate antiinflammatory functions during experimental allergic airway inflammation. JCI Insight. 2019;4:e123216. 10.1172/jci.insight.123216 PMC6413774 30674714

[33] Xu F , Yi J , Wang Z , et al. IL‐27 regulates the adherence, proliferation, and migration of MSCs and enhances their regulatory effects on Th1 and Th2 subset generations. Immunol Res. 2017;65:903‐912. 28612255 10.1007/s12026-017-8929-8 PMC5544780

[34] Kumar P , Rajasekaran K , Nanbakhsh A , Gorski J , Thakar MS , Malarkannan S . IL‐27 promotes NK cell effector functions via Maf‐Nrf2 pathway during influenza infection. Sci Rep. 2019;9:4984. 30899058 10.1038/s41598-019-41478-6 PMC6428861

[35] Jung J‐Y , Roberts LL , Robinson CM . The presence of interleukin‐27 during monocyte‐derived dendritic cell differentiation promotes improved antigen processing and stimulation of T cells. Immunology. 2015;144:649‐660. 25346485 10.1111/imm.12417 PMC4368171

[36] da Silva MB , da Cunha FF , Terra FF , Camara NOS . Old game, new players: Linking classical theories to new trends in transplant immunology. World J Transplant. 2017;7:1‐25. 28280691 10.5500/wjt.v7.i1.1 PMC5324024

[37] Borges TJ , O'Malley JT , Wo L , et al. Codominant role of interferon‐γ‐ and interleukin‐17‐producing T cells during rejection in full facial transplant recipients. Am J Transplant. 2016;16:2158‐2171. 26749226 10.1111/ajt.13705 PMC4979599

[38] Collison LW , Delgoffe GM , Guy CS , et al. The composition and signaling of the IL‐35 receptor are unconventional. Nat Immunol. 2012;13:290. 22306691 10.1038/ni.2227 PMC3529151

[39] Jiang Y , Wang J , Li H , Xia L . IL‐35 alleviates inflammation progression in a rat model of diabetic neuropathic pain via inhibition of JNK signaling. J Inflamm. 2019;16:19. 10.1186/s12950-019-0217-z PMC6651949 31367192

[40] Ma N , Fang Y , Xu R , et al. Ebi3 promotes T‐ and B‐cell division and differentiation via STAT3. Mol Immunol. 2019;107:61‐70. 30660991 10.1016/j.molimm.2019.01.009

[41] Liu KS , Fan XQ , Zhang L , et al. Effects of recombinant human interleukin‐10 on Treg cells, IL‐10 and TGF‐β in transplantation of rabbit skin. Mol Med Rep. 2014;9:639‐644. 24270972 10.3892/mmr.2013.1817 PMC3896515

[42] Moreau A , Varey E , Anegon I , Cuturi M‐C . Effector mechanisms of rejection. Cold Spring Harb Perspect Med. 2013;3:a015461. 24186491 10.1101/cshperspect.a015461 PMC3808773

[43] Gaignage M , Marillier RG , Cochez PM , et al. The TLR7 ligand R848 prevents mouse graft‐versus‐host disease and cooperates with anti‐interleukin‐27 antibody for maximal protection and regulatory T‐cell upregulation. Haematologica. 2019;104:392‐402. 30213828 10.3324/haematol.2018.195628 PMC6355498

